# Antihyperalgesic Properties of Honokiol in Inflammatory Pain Models by Targeting of NF-κB and Nrf2 Signaling

**DOI:** 10.3389/fphar.2018.00140

**Published:** 2018-03-20

**Authors:** Sidra Khalid, Muhammad Z. Ullah, Ashraf U. Khan, Ruqayya Afridi, Hina Rasheed, Adnan Khan, Hussain Ali, Yeong S. Kim, Salman Khan

**Affiliations:** ^1^Department of Pharmacy, Faculty of Biological Sciences, Quaid-i-Azam University, Islamabad, Pakistan; ^2^College of Pharmacy, Seoul National University, Seoul, South Korea

**Keywords:** honokiol, allodynia, hyperalgesia, CFA, carrageenan, cytokines

## Abstract

The present study investigates the possible anti-nociceptive effect of intraperitoneal (i.p.) honokiol: a phenolic compound originally isolated from *Magnolia officinalis*, in acute and chronic inflammatory pain models. Doses of 0.1, 5, and 10 mg/kg honokiol were administered in carrageenan induced pain and the dose (honokiol 10 mg/kg i.p.) with most significant response among behavioral tests was selected for further experiments. The i.p. administration of honokiol inhibits mechanical hyperalgesia, mechanical allodynia, and thermal hyperalgesia, without causing any apparent toxicity. To elucidate the effect of honokiol on various cytokines and antioxidant enzymes, quantitative real-time-PCR was performed to determine the expression levels of pro-inflammatory cytokines and antioxidant enzymes. It is demonstrated that honokiol significantly reduced the expression levels of tumor necrosis factor (TNF-α), interleukin-1β (IL-1β), interleukin-6 (IL-6), and vascular endothelial growth factor (VEGF). Similarly, honokiol was also found to potentiate the expression of nuclear factor erythroid 2–related factor 2 (Nrf2), superoxide dismutase 2 (SOD2), and heme oxygenase-1 (HO-1) levels. Additionally, honokiol significantly reduced plasma nitrite levels as compared to complete Freund’s adjuvant (CFA) induced group. X-ray analysis and hematoxylin and eosin (H&E) staining of inflamed and treated paws showed that honokiol reduced the inflammation with significantly less leukocyte infiltration and soft tissue inflammation. In order to explore the possible mechanism of action of honokiol, agonists [piroxicam (5 mg/kg), tramadol (50 mg/kg), and gabapentin (5 mg/kg) i.p.] as well as antagonists [naloxone (4 mg/kg), olanzapine (10 mg/kg), and flumazenil (0.2 mg/kg) i.p.] were used to study involvement of various receptors on the anti-nociceptive effect of honokiol. The potential side effects of honokiol on muscle activity were assessed. An adverse effect testing of honokiol by liver and renal functions were also carried out. The effect of oral honokiol was also assessed on gastrointestinal (GIT) mucosa. Our results demonstrate that honokiol has a significant anti-nociceptive activity through inhibition of anti-inflammatory mediators.

## Introduction

Pain serves as an alarm against potential or actual tissue damage and initiates an appropriate protective response (Kidd and Urban, 2001). Pain may extend beyond its protective role and become persistent in damaged and inflamed tissue (Stein et al., 2009). Tissue inflammation stimulates cytokines and enzymes responsible for inflammatory pain. During the inflammatory process the release of chemical mediators (e.g., NO, TNF-α, IL-1β, IL-6 and growth factors), leads to nociceptor sensitization (Fein, 2012). This sensitization of primary nociceptor leads to dysesthesia, a disagreeable and abnormal sensation. Likewise the sensitization of nociceptors is also the major denominator of all the inflammatory pains that lead to the state of hyperalgesia and allodynia (Basbaum et al., 2009). This sensitization of nociceptors lowers the neuronal threshold by activation of numerous signaling pathways such as NF-κB, CREB, MAPKs, and various other transcription factors (Strong et al., 2002). Clinically, pain sensitization can be classified as hyperalgesia: an augmented response to noxious stimuli and allodynia, i.e., pain in response to a stimulus that normally fails to provoke pain (Julius and Basbaum, 2001).

The development of new treatment options for treatment of pain is particularly challenging (Locatelli et al., 2017) Although, a number of drugs are available such as non-steroidal anti-inflammatory drugs (NSAID), opioids, and antiepileptic, these treatments are often accompanied by dose limiting side effects (Rainsford, 2007). The anti-inflammatory herbal drugs could represent an innovative source of analgesic (Lim et al., 2014). The herbal drugs could be more efficient, cost effective, and safe is still highly desirable (Parenti et al., 2016). Honokiol, a polyphenol with low molecular weight was isolated from the bark of *Magnolia officinalis*, exhibits a number of pharmacological properties that demonstrate anti-cancer (Liu et al., 2008; Fried and Arbiser, 2009), anti-inflammatory (Lin et al., 2009), and hepatoprotective (Park et al., 2003) activities. Honokiol has known anti-inflammatory activity through inhibition of protein kinase C, mitogen-activated protein kinase, and other multiple pathways (Kim and Cho, 2008; Chao et al., 2010). Given its potent pharmacological properties, it is hypothesized that honokiol might be a promising candidate as a new treatment for pain. The current study examined the effect of honokiol in various pain models in mice.

## Materials and Methods

### Animals

All of the experiments were carried out in adult male BALB/c mice weighing 20–30 g according to procedures described previously. Animals’ were housed under controlled environmental conditions (23 ± 1°C, 55 ± 5% humidity and a 12-h light/dark cycle) and maintained with free access to water and a standard laboratory diet. All experiments were performed in accordance with the “Principles of Laboratory Animal Care” from the NIH. Similarly, the experimental procedures mentioned in current guidelines for the laboratory animals care and the ethical guidelines for conduction of experimental pain in conscious animals as previously specified (Zimmermann, 1983) were followed. Behavioral experiments were performed between 8 am and 6 pm. Each group contained five mice. All the animals were used only for one time. Behavioral experiments were performed between 8 am to 6 pm.

Doses of 0.1, 5, and 10 mg/kg honokiol were administered in carrageenan (100 μl/paw) induced pain and the dose (honokiol: 10 mg/kg i.p.) with most significant response in behavioral tests was selected for further experiments. The animals were divided into four groups in case of chronic model of CFA [Normal; saline with 2% Dimethyl sulfoxide (DMSO), negative control; saline with 2% DMSO only, 5 mg/kg Dexa, 10 mg/kg i.p. honokiol dissolved in normal saline and 2% DMSO]. In mechanistic study of pain induced by carrageenan there were fifteen groups (Normal; saline with 2% DMSO, negative control; saline with 2% DMS, 10 mg/kg i.p. honokiol, 5 mg/kg gabapentin, 50 mg/kg tramadol, 5 mg/kg piroxicam, 4 mg/kg naloxone, 10 mg/kg olanzapine, 0.2 mg/kg flumazenil either alone or in combination with honokiol). All procedures were complied with “Animal care guidelines of QAU” Islamabad. This study was approved by Bio-ethical Committee (Approval No. BEC-FBS-QAU2017-14) of Quaid-i-Azam University and protocol number was assigned. All the experiments were designed to cause minimum harm to animals. The bioethical certificate is attached here with the MS.

### Reagents and Drugs

To induce cutaneous inflammation CFA (Sigma–Aldrich, Germany), was injected into the plantar surface of one hind paw (20 μl/paw). Carrageenan (Sigma–Aldrich, Germany) (100 μl/paw) was administered in separate cohorts of mice as an additional model of cutaneous tissue injury and inflammation, griess reagent, gabapentin (5 mg/kg), tramadol (50 mg/kg), piroxicam (5 mg/kg) (Merck, Darmstadt, Germany), dexamethasone (5 mg/kg), chloroform, distilled water, 2% DMSO were of research grade. Honokiol was isolated as previously described (Park et al., 2009) (10 mg/kg i.p.) dissolve in 2% DMSO and normal saline was administered before CFA or carrageenan induced inflammation. Elisa kits of TNF-α and IL-1β (Thermo Fisher Scientific, United States), NF-κB (p65) (Cayman Chemical, Ann Arbor, MI, United States).

### Dose Selection

In a set of experiment the dose response of honokiol at the doses of 0.1, 5, and 10 mg/kg against carrageenan (100 μl/paw) was assessed. Treatments were provided 45 min before carrageenan injection. For dose-response determination mice were allocated to experimental groups: (A) Normal, (B) Carrageenan, (C) Piroxicam (10 mg/kg), (D) Carrageenan+honokiol 0.1 mg/kg, (E) Carrageenan+honokiol 5 mg/kg, and (F) Carrageenan+honokiol 10 mg/kg. After 4 h of carrageenan injection, the dose at which most significant activity was evident against mechanical allodynia, hyperalgesia, thermal hyperalgesia and effect against paw edema was chosen for further experiments.

### Behavioral Tests

#### Assessment of Mechanical Allodynia

To evaluate mechanical allodynia induced by carrageenan or CFA, the mechanical testing procedure was modified from previously described methods (Schäfers et al., 2003). Mice were given a 1 week habituation period prior to baseline testing. On the day of testing mice were placed in sufficiently sized porous mesh metal cages to habituate for 30 min before threshold testing. Mechanical thresholds were assessed using a series of calibrated monofilaments (Von Frey; Stoelting, Kiel, WI, United States) at three different time points (2, 4, and 6 h) after the induction of hind paw inflammation with CFA or carrageenan. Mice were placed on an elevated wire grid and the plantar surface of the paw was stimulated with von Frey hair monofilaments using a range of ascending forces. The lowest force that evoked an abrupt withdrawal response was considered as a threshold. The force at which the paw withdrawal occurred was recorded (Khan et al., 2014). For the investigation of long-term treatment effects, mechanical sensitivity was evaluated 4 h after CFA injection for 0–6 days to determine the tolerance effect (Costigan et al., 2009).

#### Mechanical Hyperalgesia

For the assessment of mechanical hyperalgesia, a digital Randall Selitto test was used following previously described methodology (de Oliveira et al., 2011). The end-point was the clear paw movement or the paw withdrawal in response to the pressure applied to the hind paw by a hand held transducer.

#### Thermal Hyperalgesia

Thermal hyperalgesia was investigated using the hot plate test with slight modifications (Hijazi et al., 2017). Animals were individually placed on the hot plate (Ugo Basile, Varese, Italy) at a temperature set to 55°C. Heat exposure was continued until pain response of CFA treated hind paw occurred. The animals were tested at 2, 4, and 6 h after CFA injection for the 1 day and then at 4 h after daily treatment with honokiol or vehicle. Testing was carried out daily up to 6 days. The end-point was the characteristic hind paw shaking and/or withdrawal. The paw withdrawal latency was documented manually. To avoid tissue damage, mice were given a maximum time permitted on the hot surface of 35 s (Hijazi et al., 2017).

#### Cold Pain

The cold pain was evaluated using acetone drop method as described previously (Khan et al., 2016), with necessary modification. A drop of acetone (25 μl), was precisely administered onto the lateral plantar surface of the hind paw, using a micropipette without touching the skin. The duration of the withdrawal response was recorded with an arbitrary minimal value of 0.5 s and a maximum cutoff time of 20 s (Choi et al., 1998).

### Analysis of Possible Mechanisms of Action

To investigate the possible anti-nociceptive mechanism of action of honokiol (10 mg/kg) a study was conducted as described previously (Quintão et al., 2012) with slight modifications. Mice pretreated with carrageenan (100 μl/paw) were administered with different types of agonists and antagonist either alone or in combination with honokiol to assess the involvement of various receptors. Mice were pretreated with the cyclooxygenase inhibitor, piroxicam (5 mg/kg i.p.); gabapentin (50 mg/kg i.p.); or the non-selective opioid receptor agonist, tramadol (50 mg/kg, i.p.) each, either alone or co-administered with honokiol (10 mg/kg i.p.). To investigate the involvement of opioid receptor the non-selective opioid receptor antagonist naloxone (2.5 μmol/kg i.p.) was administered to group of five mice. The involvement of GABA was evaluated by treating mice with an antagonist of the benzodiazepine receptors, flumazenil (0.2 mg/kg i.p.). To assess the participation of the dopaminergic or GABAergic systems, another group of mice received olanzapine (10 mg/kg i.p.), a non-selective dopaminergic antagonist. After pre-treatment with the antagonists (15 min), mice were injected with honokiol (10 mg/kg i.p.), and after 4 h the hypernociception was evaluated. Mice were tested (von Frey, Randall Selitto, hot plate) 4 h after treatments (Khan et al., 2014).

### Effect of Honokiol on Paw Edema

Paw thickness was measured by the dial thickness gauge. One hour before the induction of cutaneous tissue inflammation mice were treated with normal saline (2% DMSO) in the normal control group, Dexa (5 mg/kg) in the positive control group and honokiol (10 mg/kg) in the treatment group. The negative control group was injected just with CFA and no treatment was provided to them. Paw edema was evaluated at 2, 4, and 6 h for acute effects and at 0–6 days for chronic effects. Day 5 was excluded from treatment to assess the possible tolerance effect of the drug.

### Effect of Honokiol on Muscle Coordination

To investigate the nonspecific effect of honokiol (10 mg/kg) on locomotor coordination locomotor activity was assessed 6 h following CFA induced inflammation (acute assessment) and at 6 days after chronic treatment with honokiol. Muscle coordination was assessed using Kondziela’s inverted screen test and the weight lifting test (Deacon, 2013). For the inverted screen test, the mice were acclimatized in the experiment room 5–20 min beforehand. Mice were placed in the middle of the wire mesh screen and the screen was rotated into an inverted position after starting a stopwatch. The screen was held stable 40–50 cm above a soft surface to avoid injury in case of falls. The time taken before a mouse falls off the inverted screen was recorded. A cutoff of 60 s was used. Mice were scored based on the following scale: falls occurring between 1 and 10 s were scored as 1, between 11 and 25 s as 2, falling after 26–59 s as 3 and mice reaching the cutoff time of 60 s = 4.

Additionally, the weight lifting test was performed. Multiple standardized weights were used as described previously (Deacon, 2013). A total final score was calculated as the number of links in the heaviest chain held for a full 3 s, multiplied by the time (s) it was held. If the mice failed to lift the heaviest weight for 3 s then the appropriate intermediate value was calculated (Deacon, 2013).

### Biochemical Assays

#### Nitric Oxide (NO) Determination

Nitric oxide production from plasma was analyzed using the Griess reagent method. Mice were sacrificed after 6 days of treatment with excess CO_2._ Blood was collected in EDTA tubes and centrifuged at 5000 rpm for 10 min. For NO analysis plasma was obtained, 50 μl of blood plasma was mixed with 50 μl of saline and then mixed with 100 μl of Griess reagent. After 30 min of incubation at room temperature NO concentration was determined as described previously (Tsikas, 2007).

#### RNA Extraction and Reverse Transcriptase (RT)-PCR

The mRNA levels of the various target genes (TNF-α, IL-1β, IL-6, Nrf2, HO-1, SOD2, VEGF, and GAPDH) were determined. Levels of the target genes in hind paw skin (inflamed or not inflamed) were assessed using qRTPCR using methods as previously described (Khan et al., 2014). Briefly, 48-well plates were used for quantitative PCR. 10 pmol primers (forward and reverse), and the working solution SYBR green were used under the following conditions: heat for 5 min at 95°C, 1 min of 40 cycles of 95°C, then 55°C for 45 s, and finally 72°C for 30 s. The optimal conditions, melting point, and the reaction specificity were determined beforehand. The sequences of the PCR primers are outlined in **Table 1**. As an internal standard to control amplification variability due to changes in starting mRNA concentrations, a housekeeping gene, GAPDH, was chosen. For each transcript, the copy number was calculated relative to the GAPDH normalized copy number.

**Table 1:** The sequences of the PCR forward and reverse primers and amplification temperature.

S. No.	Gene	Forward primer	Reverse primer	°C
1	GAPDH	TGCACCACCAACTGCTTAGC	GGCATGGACTGTGGTCATGAG	55
2	HO-1	CACGCATATACCCGCTACCT	CCAGAGTGTTCATTCGAGA	58
3	SOD2	GCGGTCGTGTAAACCTCAT	CCAGAGCCTCGTGGTACTTC	58
4	Nrf2	CCTCGCTGGAAAAAGAAGTG	GGAGAGGATGCTGCTGAAAG	55
5	IL-1β	AGAAGCTTCCACCAATACTC	AGCACCTAGTTGTAAGGAAG	55
6	IL-6	GCCCTTCAGGAACAGCTATGA	TGTCAACAACATCAGTCCCAAGA	60
7	TNF-α	CTTCTCCTT CCT GAT CGT GG	GCT GGT TAT CTC TCA GCT CCA	62
8	VEGF	CAAGACAAGAAAATCCCTGTGG	CCTCGGCTTGTCACATCTG	55


#### TNF-α, IL-1β, and NF-κB (p65) Production by ELISA Kits

The tissue samples from all the groups (vehicle, negative, Dexa, and honokiol (10 mg/kg i.p.) were determined using a commercially available ELISA kits. TNF-α and IL-1β (eBioscience, Inc., San Diego, CA, United States) and NF-κB (p65) (Cayman Chemical, Ann Arbor, MI, United States) kits were used as per manufacturer instructions. For sample preparation the paw skin tissue was removed, tissue protein extraction was done by using Phosphate buffer saline (PBS) in concentration of 100 mg tissue/ml PBS, to which 0.4 M NaCl, 0.05%, Tween 20, and protease inhibitors were added. The prepared sample was then centrifuged at 3000 *g* for 10 min as method described previously (Khan et al., 2013b).

#### X-Ray Analysis

X-rays were used to investigate bone morphology and surrounding soft tissues inflammation induced by CFA. Control mice were treated only with CFA (20 μl/paw). Animals were sacrificed after 6th day of treatment and paws were examined by the X-ray radiography and software for visualization (Chen et al., 2012).

#### Histological Analysis

Hematoxylin and eosin staining was performed as described (Duerr, 2006). Briefly, the skin from the inflamed paw was removed and washed with normal saline, fixed in 10% formalin. Water was removed by dipping in alcohol and then the tissue was embedded in paraffin according previously described methodology (Yen et al., 2009). Paw tissue was sectioned at 4 μm thickness, stained with hematoxylin and eosin and visualized with a light microscope.

#### Analysis of Liver and Kidney Enzymes in Blood Plasma

Liver and kidney enzymes such as aspartate aminotransferase (AST) and alanine aminotransferase (ALT) and creatinine were analyzed. The test was performed on blood plasma, collected on the final day of the experiment. Blood was collected from cardiac puncture and centrifuged at 5000 rpm for 10 min. Standardized kits were used for testing. For the photometric determination of serum enzyme levels, commercial kits (ALT, AST activity assay kits of Merck, France), creatinine (Live diagnostic Inc., Canada) were used.

#### Effect of Honokiol on Histopathology of GIT Mucosa

Male albino mice, weighing 22–30 g were sorted randomly in three groups, normal, piroxicam (10 mg), and honokiol (10 mg/kg oral). All the mice were fasted for 24 h before the study. Mice were administered with piroxicam (10 mg/kg per oral) and honokiol (10 mg/kg oral) by metal orogastric tube for 3 days by the method described else were with modifications (Brzozowskia et al., 2000). Histological evaluation of gastric mucosa was done to evaluate the effect of drugs on stomach mucosal integrity.

### Statistical Analysis

Results, unless otherwise stated, are expressed as the means ± standard deviations (SD) from three individual experiments. For each group five animals were randomly assigned and average is taken. One way analysis of variance (ANOVA), along with Dunnett’s *t*-test was used to determine statistical significance between groups. The “*p*” value less than 0.05 was considered as statistically significant.

## Results

### Dose Selection and Optimizations

**FIGURE 1:**
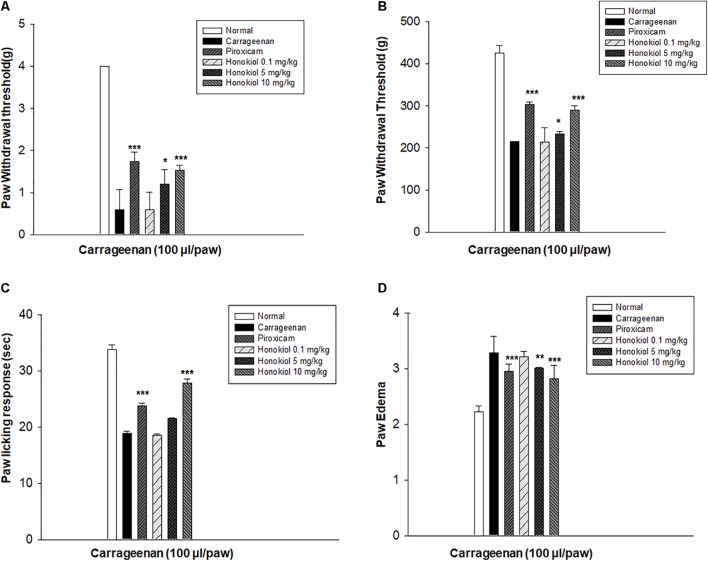
The dose response of honokiol at the doses of 0.1, 5, and 10 mg/kg i.p. against carrageenan (100 μl/paw)-induced inflammatory model in mice. For dose response determination, mice were allocated to experimental groups: normal, carrageenan, piroxicam (5 mg/kg i.p.) and carrageenan+honokiol i.p. 0.1 mg/kg, carrageenan+honokiol i.p. 5 mg/kg, and carrageenan+honokiol i.p. 10 mg/kg. After 4 h of carrageenan administration, **(A)** mechanical allodynia, **(B)** mechanical hyperalgesia, **(C)** thermal hyperalgesia, and **(D)** anti-inflammatory effects against paw edema were recorded. The data obtained are represented as the means (*n* = 5) ± SD, ^∗^*P* < 0.05, ^∗∗^*P* < 0.01, and ^∗∗∗^*P* < 0.001 points out the significant differences from the negative control: carrageenan induced group. ^###^indicates a significant difference vs. negative control group.

In order to select the most effective dose of honokiol, carrageenan-induced acute model of inflammation was performed. Honokiol at the doses of 0.1, 5, and 10 mg/kg were selected for the study. The results showed that honokiol at dose 0.1 and 5 mg/kg didn’t produce any significant anti-inflammatory, anti-hyperalgesic, and anti-allodynic effects against carrageenan-induced inflammatory model. It was observed that maximum concentration (10 mg/kg) produced very significant response against inflammation and pain as shown in **Figure 1**. Therefore, honokiol (10 mg/kg) was selected for further testing.

### Effect of Honokiol on CFA-Induced Mechanical Allodynia

**FIGURE 2:**
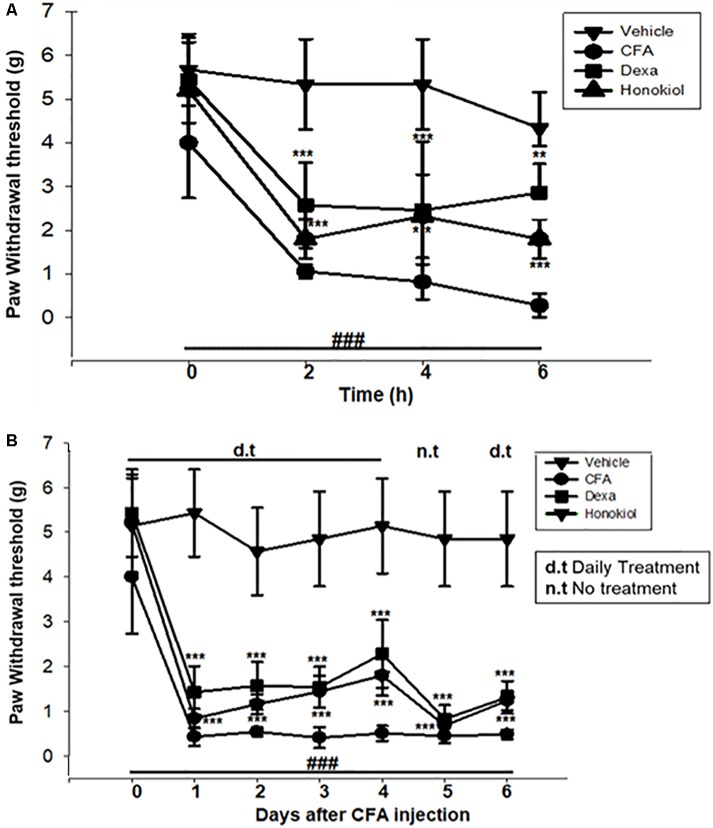
**(A)** Effect of honokiol (10 mg/kg) treatment on acute allodynia **(B)** Effect of honokiol treatment on chronic allodynia. The data obtained are represented as the means (*n* = 5) ± SD, ^∗^*P* < 0.05, ^∗∗^*P* < 0.01, and ^∗∗∗^*P* < 0.001 points out the significant differences from the CFA-induced group. ^###^indicates a significant difference vs. negative control group.

The i.p. administration of honokiol (10 mg/kg) caused a significant inhibition of CFA-induced mechanical allodynia. Paw withdrawal thresholds after 2, 4, and 6 h of CFA (20 μl/paw) as shown in **Figure 2A**. Dexa (5 mg/kg i.p.), a positive control, also produced a significant inhibition of the CFA-induced allodynic responses. Similarly, it was notable that the anti-nociceptive effect was significant in case of the chronic model, specialy at day 6. The results showed that honokiol remarkably reduced pain response throughout the chronic inflammatory pain model (**Figure 2B**).

#### Effect of Honokiol on CFA-Induced Mechanical Hyperalgesia

**FIGURE 3:**
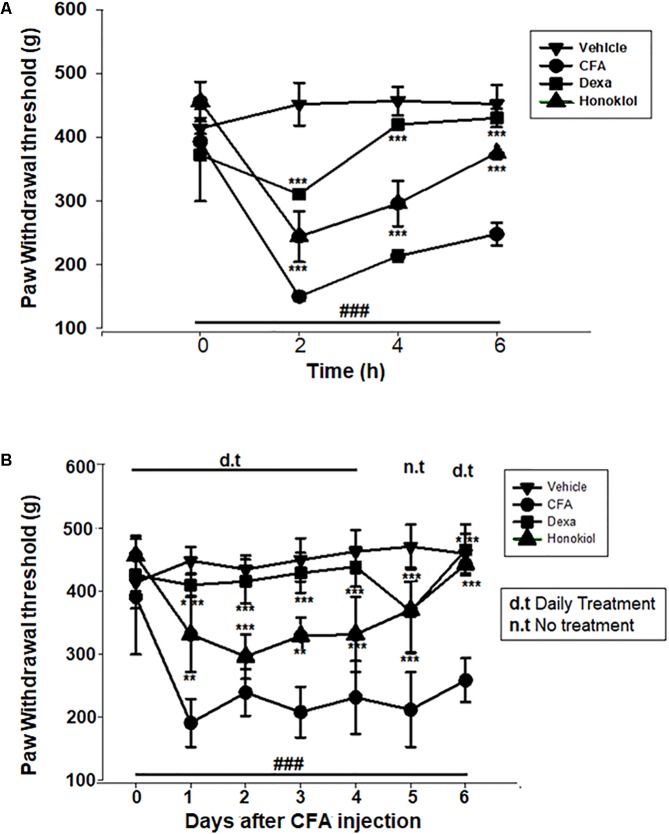
**(A)** Inhibition of CFA-induced mechanical hyperalgesia was measure every 2 h after CFA (20 μl/paw) injection. **(B)** Anti-nociceptive effect on honokiol (10 mg/kg) on CFA-induced mechanical hyperalgesia. Effect on mechanical hyperalgesia was measured every day in CFA treated mice, from 0 to 6 days with an interval of day five in order to construe the possible tolerance effect. The data are reported as the means (*n* = 5) ± SD, ^∗^*P* < 0.05, ^∗∗^*P* < 0.01, and ^∗∗∗^*P* < 0.001 indicate significant differences from the CFA-treated group. ^###^indicates a significant difference vs. negative control group.

The anti-nociceptive activity of honokiol (10 mg/kg) on CFA and carrageenan induced mechanical hyperalgesia was investigated. Pre-treatment with both honokiol (10 mg/kg i.p.) and the reference drug Dexa (5 mg/kg i.p.) significantly inhibited mechanical hyperalgesia. In the acute phase, as depicted in **Figure 3A** the highest pain threshold was exhibited at 6 h. After chronic treament with honokiol, the highest threshold of mechanical hyperalgesia was at day 6 (**Figure 3B**). There was a clear dip in nociceptive activity at the no treatment day indicating no tolerance effect.

#### Effect of Honokiol on CFA-Induced Thermal Hyperalgesia (Hot)

**FIGURE 4:**
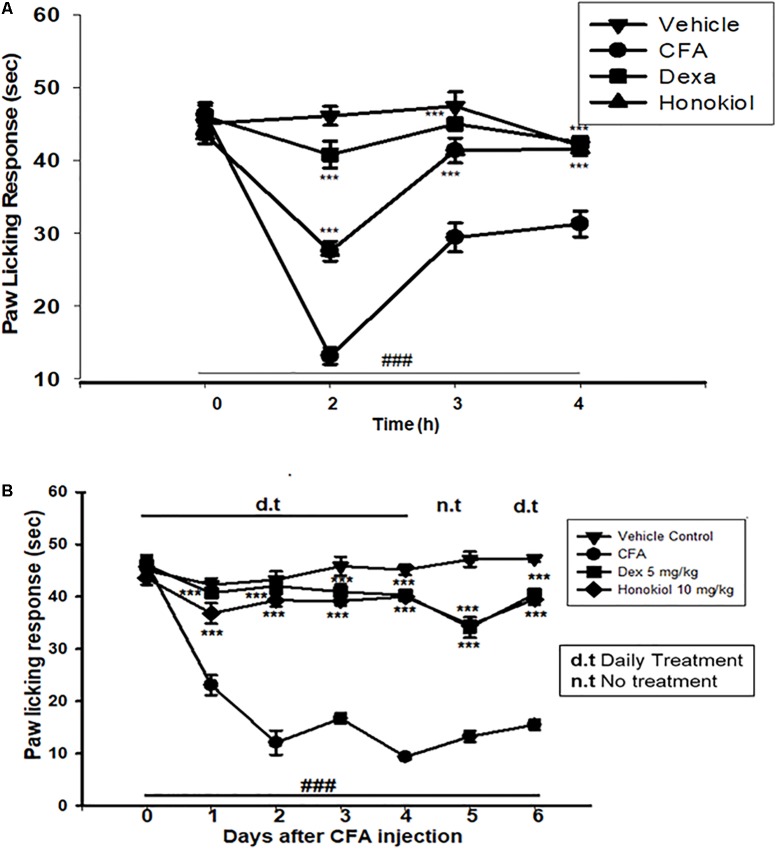
**(A)** Inhibition of CFA-induced thermal hyperalgesia at 0, 2, 4, and 6 time points due to trreatment of honokiol (10 mg/kg i.p.). **(B)** Chronic effect of honokiol on thermal hyperalgesia was measured every day after CFA injection from 0 to 6 days with an interval of day five in order to interpret the possible tolerance effect. The data are reported as the means (*n* = 5) ± SD, ^∗^*P* < 0.05, ^∗∗^*P* < 0.01, and ^∗∗∗^*P* < 0.001 indicate significant differences from the CFA-treated group. ^###^*P* < 0.001 indicates a significant difference from the control group.

Honokiol (10 mg/kg) also produced a significant inhibition of CFA-induced thermal hyperalgesia after 2, 4, and 6 h after CFA induced inflammation (**Figure 4A**). Dexa (5 mg/kg i.p.), significantly inhibited CFA-induced thermal hyperalgesia. It was noteworthy that the analgesic effect was most pronounced at 6 h (*P* < 0.01). Chronic treatment with honokiol reduced thermal hyperalgesia as compare to vehicle treated controls especially at day 4 with the inhibition of paw licking response up to 40 ± 0.7 s. At day 5 (no treatment day) there was clear reduction in activity showing no tolerance effect (**Figure 4B**).

#### Effect of Honokiol on CFA-Induced Cold Allodynia

**FIGURE 5:**
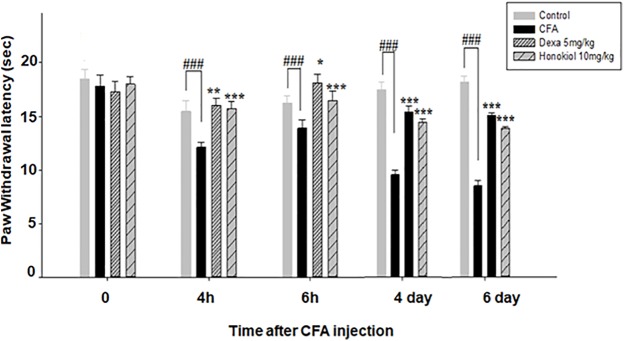
Effect of honokiol (10 mg/kg i.p.) on CFA-induced thermal hyperalgesia (cold) induced by acetone. The acute effect was measured after 4 and 6 h and inhibition of CFA-induced thermal hyperalgesia (cold) measured after 4 and 6 days of CFA injection to evaluate the chronic effect. The data were reported as the means (*n* = 5) ± SD, ^∗^*P* < 0.05, ^∗∗^*P* < 0.01, and ^∗∗∗^*P* < 0.001 indicate significant differences from the CFA-treated group. ^###^indicates a significant difference vs. negative control group.

Honokiol inhibited inflammation induced cold allodynia at 4 and 6 h (*P* < 0.001) (**Figure 5**). To evaluate the long-term therapeutic effect of honokiol, another set of experiments was carried out. As established previously, mice were treated once a day for 6 days with honokiol (10 mg/kg) (**Figure 5**). Chronic treatment with honokiol prevented inflammation induced cold allodynia. Vehicle treatment did not affect hyperalgesia, while the positive control drug Dexa inhibited CFA-induced hyperalgesia remarkably.

#### Effect of Honokiol on CFA-Induced Paw Edema

**FIGURE 6:**
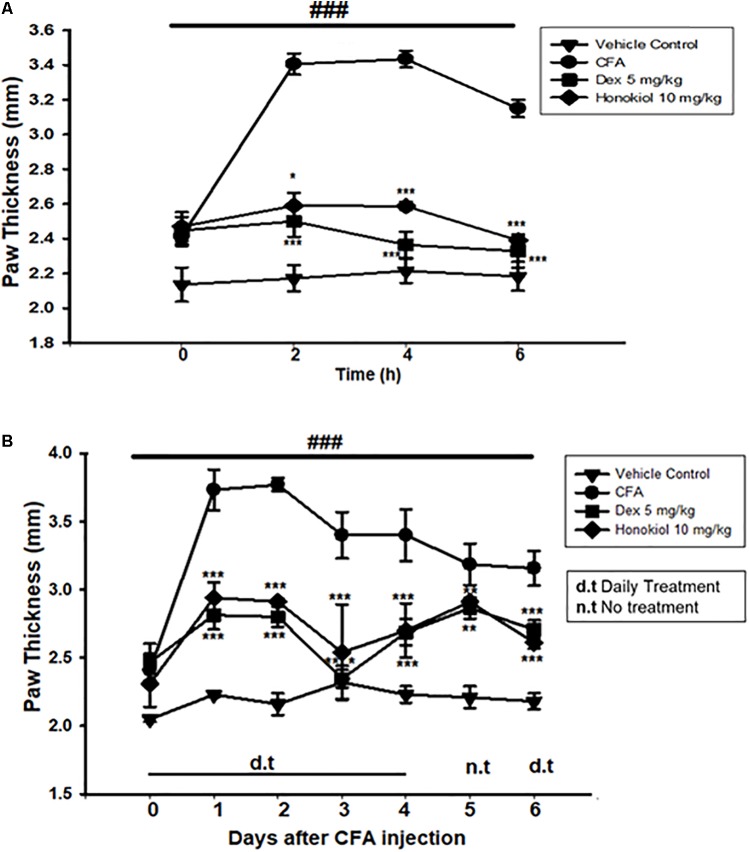
**(A)** Effect of honokiol on CFA-induced paw edema. The withdrawal of paw was measured every 2 h after CFA pre-treatment, up to 6 h. **(B)** Effect of honokiol on CFA-induced chronic paw edema up to 6 days as explained in the “Materials and methods” section. The paw withdrawal effect was accessed every day up to 6 days. The data are reported as the means (*n* = 5) ± SD, ^∗^*P* < 0.05, ^∗∗^*P* < 0.01, and ^∗∗∗^*P* < 0.001 show significant differences from the CFA-treated negative control group. ^###^*P* < 0.001 indicates a significant difference from the control group.

To explore the link between the anti-nociceptive and anti-inflammatory effect of honokiol, CFA-induced paw edema was assessed. Administration of honokiol (10 mg/kg) 1 h before CFA significantly reduced paw edema at 4 and 6 h after the induction of inflammation (**Figure 6A**). Dexa (5 mg/kg) also significantly inhibited CFA-induced edema. The reduction in paw edema was observed for up to 6 days after CFA (**Figure 6B**).

#### Possible Mechanism of Action in Carrageenan-Induced Inflammatory Pain Responses

**FIGURE 7:**
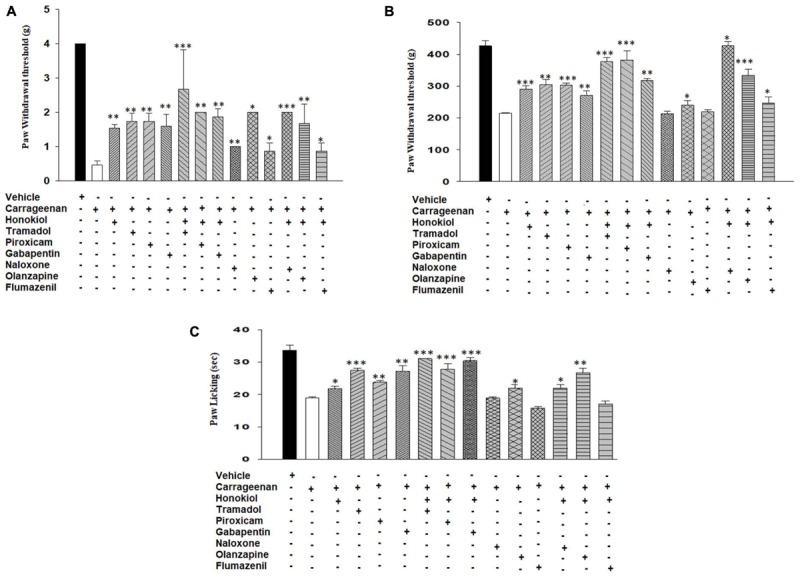
Effects of pre-treatment with agonists piroxicam (5 mg/kg i.p.), tramadol (50 mg/kg i.p.), and gabapentin (50 mg/kg i.p.) and antagonists naloxone (2.5 μmol/kg), olanzapine (10 mg/kg) and flumazenil (0.2 mg/kg) on the anti-nociceptive effects induced by honokiol (10 mg/kg, i.p.) in the carrageenan (100 μl/paw) induced inflammatory pain model. **(A)** Effect on carrageenan-induced mechanical allodynia. **(B)** Effect on carrageenan-induced mechanical hyperalgesia. **(C)** Effect on carrageenan-induced thermal hyperalgesia. The data are reported as the means (*n* = 5) ± SD, ^∗^*P* < 0.05, ^∗∗^*P* < 0.01, and ^∗∗∗^*P* < 0.001 show significant differences from the CFA-treated negative control group.

To elucidate the possible mechanism of action, the mechanistic study was performed. Results were illustrated in **Figure 7** showing that pre-treatment of animals with naloxone or flumazinil before the injection of honokiol significantly reversed the anti-nociceptive effects of honokiol in mice injected with carrageenan. In contrast, pre-treatment of animals with olanzapine did not interfere with the effects of honokiol, tramadol, and gabapentin potentiate the effect of honokiol (**Figures 7A–C**).

#### Honokiol Effect on Nitric Oxide Production in Blood Plasma

**FIGURE 8:**
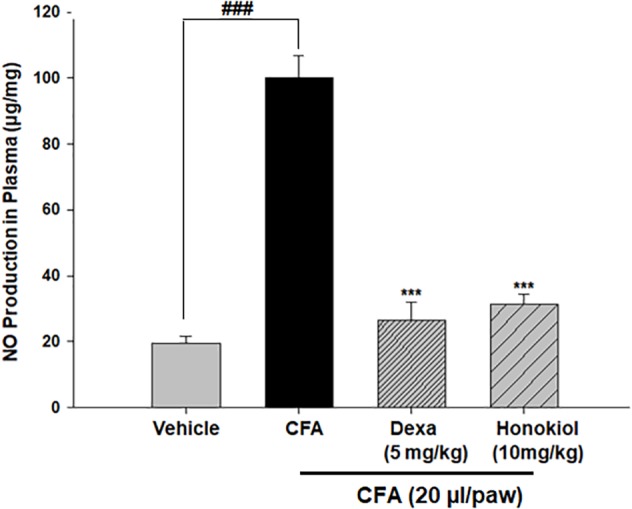
Effect of honokiol on inhibition of nitric oxide (NO) in CFA induced honokiol (10 mg/kg) treated paw tissue. Each group represents the mean of five animals. Error bar shows the standard deviation. ^∗^*P* < 0.05, ^∗∗^*P* < 0.01, and ^∗∗∗^*P* < 0.001; significantly different from negative control’ mechanical threshold: ^###^*P* < 0.001.

The production of nitrate in blood plasma after CFA-induced inflammation was investigated 6 days after CFA injection (**Figure 8**). CFA-induced inflammation significantly increased the production of NO (100 ± 6 ug/ml). Honokiol significantly reduced NO production after 6 days of treatment (31.6 ± 3 ug/ml).

#### Effect of Honokiol on Pro-inflammatory Cytokines (IL-6, IL-1β, and TNF-α) in CFA-Induced Paw Tissue

**FIGURE 9:**
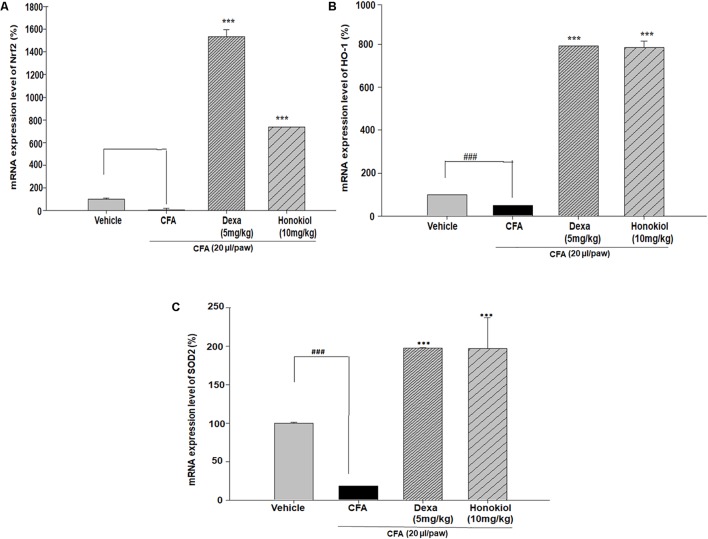
Effect of honokiol on the release of **(A)** IL-1β, **(B)** IL-6, and **(C)** on the release of pro-inflammatory cytokine TNF- α, in CFA treated paw tissue. The results were obtained from three different experiments, and expressed as mean and standard error shown as an error bar. ^∗^*P* < 0.05, ^∗∗^*P* < 0.01, and ^∗∗∗^*P* < 0.001; significantly different from negative control’ mechanical threshold: ^###^*p* < 0.001.

To explore further the anti-inflammatory effects of honokiol, the assessment of the levels of various cytokines using qRTPCR was done. The mRNA levels of IL-6, IL-1β, and TNF-α were significantly increased 6 days after CFA inflammation (**Figures 9A–C**). Honokiol significantly reduced all of these cytokines (*P* < 0.05).

#### Effects of Honokiol on TNF-α, IL-1β, and NF-κB (p65) in CFA-Induced Paw Tissue

**FIGURE 10:**
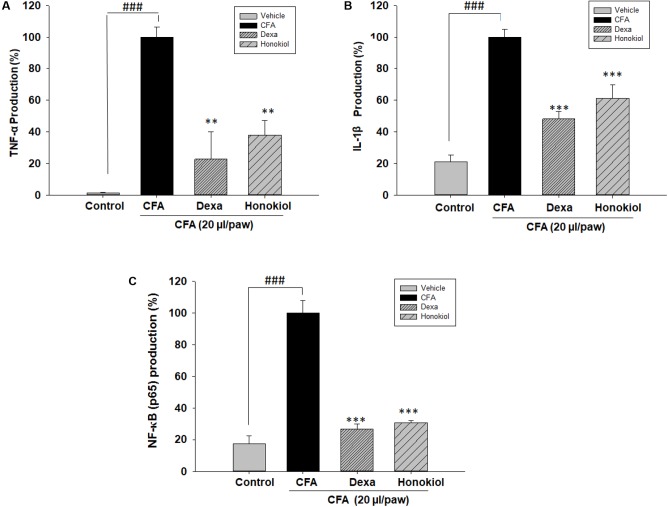
Effect of honokiol (10 mg/kg i.p.) on **(A)** TNF-α, **(B)** IL-1β, and **(C)** NF-κB (p65) production measured by ELISA. The results are shown in % age production. The data were obtained as triplicate, from each group containing five animal each, and expressed as mean and standard error shown as error bar, i.e., statistically significant difference from control values. ^∗^*P* < 0.05, ^∗∗^*P* < 0.01, and ^∗∗∗^*P* < 0.001; ^###^*P* < 0.001 indicates a significant difference from the untreated control group.

The effect of honokiol, on protein TNF-α, IL 1β, and NF-κB (p65) was investigated further by the help of Elisa kit. Production was significantly increased at 6 days after CFA induction. The increased protein levels were considerably inhibited by honokiol (**Figures 10A–C**).

#### Effect of Honokiol on Antioxidant Response Elements through Quantitative PCR, in CFA-Induced Paw Tissue

**FIGURE 11:**
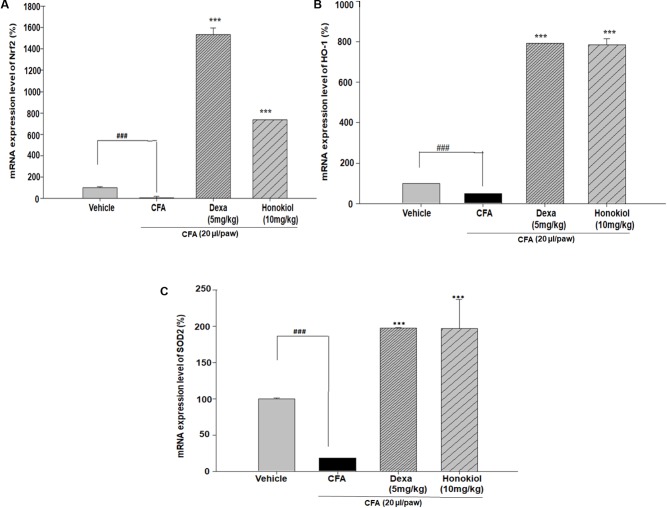
Effect of honokiol (10 mg/kg i.p.) on the gene expression levels of **(A)** Nrf2, **(B)** HO-1, and **(C)** SOD2. The results are shown in % age production. The data were obtained from three different experiments and expressed as mean and standard error shown as error bar, i.e., statistically significant difference from control values. ^∗^*P* < 0.05, ^∗∗^*P* < 0.01, and ^∗∗∗^*P* < 0.001; ^###^*P* < 0.001 indicates a significant difference from the untreated control group.

Nuclear factor erythroid 2–related factor 2, HO-1, and SOD are critical regulators of antioxidant and phase II enzymes (Sahin et al., 2010). Honokiol significantly enhanced the mRNA expression level of the antioxidants Nrf2, HO-1, and SOD (*P* < 0.05) (**Figures 11A–C**).

#### Effects of Honokiol on VEGF in CFA-Induced Paw Tissue

**FIGURE 12:**
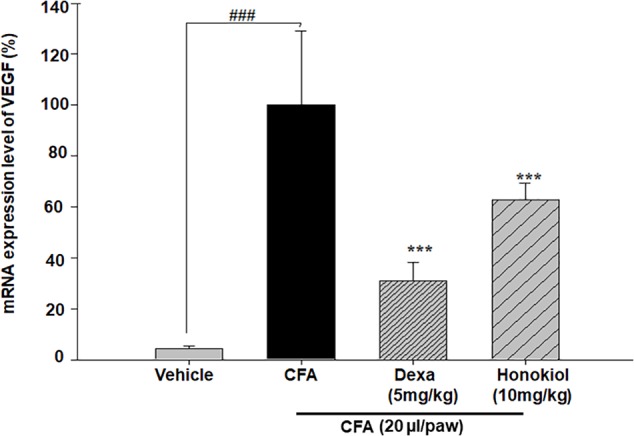
Effect of honokiol on inhibition of VEGF in CFA induced honokiol (10 mg/kg) treated paw tissue where (*n* = 5). Error bar shows the standard deviation, i.e., significant difference from control values. ^∗^*P* < 0.05, ^∗∗^*P* < 0.01 and ^∗∗∗^*P* < 0.001; ^###^*P* < 0.001 indicates a significant difference from the untreated control group.

The effect of honokiol, on signal protein VEGF was investigated further. VEGF was significantly increased at 6 days after CFA induction. The increased mRNA expression level of VEGF was considerably inhibited by honokiol (**Figure 12**).

#### Effect of Honokiol on Liver and Kidney Enzymes

To investigate the possible toxic effect of honokiol, a liver and kidney enzyme assay were performed. Treating mice with honokiol for 6 days showed no visible sign of toxicity or ill health. Obtained values, which were used as an indicator of liver and renal function, are shown in the table (**Table 2**).

**Table 2:** The effect of honokiol (10 mg/kg i.p.) on the liver and kidney functions by analyzing ALT, AST and creatinine levels.

S. No.	Group	GPT/ALT (U/L) (29.5–48.5 U/L)	GOT/AST (mg/dL) (55–87 mg/dL)	Creatinine (μmol/L)(17–70 μmol/L)
1	Normal	37.5 ± 0.5	74 ± 2	33.5 ± 1.5
2	Honokiol	60.5 ± 4.5	100.5 ± 1.5	42.5 ± 2.5


### Effect of Honokiol on X-Ray Analysis

**FIGURE 13:**
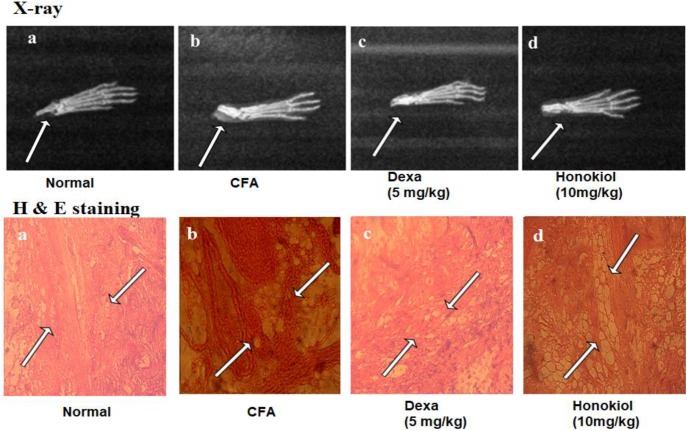
Effect of 7 days, honokiol treatment on soft tissue X-ray examination of the hind paw of mice. **(a)** Normal control **(b)** CFA induced negative control, swollen and inflamed paw of CFA treated controlled mouse compared to its normal litter mate. Note white shadow showing soft tissue inflammation **(c)** Dexamethasone (5 mg/kg) treated mice paw; **(d)** Honokiol treated mice. **(c,d)** In corresponding x-rays of dexamethasone and Honokiol, Both show significant inflammation reduction as compare to vehicle but Dexa treated paw was comparable to the normal control but there is slight soft tissue inflammation in honokiol treated group. Effect of honokiol (10 mg/kg i.p.) on CFA-mediated inflammation. H&E- hind paws of a control mouse’s stained tissue sections **(a)** Normal Mouse paw, **(b)** CFA induced mouse paw, **(c)** Mouse treated with Dexa (5 mg/kg), **(d)** Mouse treated with honokiol. Paws were analyzed on day 7 post-treatment. Each section was presented at ×4, ×10, and ×20 original magnifications. Multiple arrows indicate the location of leukocytes infiltration, large white spaces show the edema affected cells.

X-ray images (**Figure 13**) showed shadows that indicated distended soft tissue in CFA-induced mice paw. The inflamed area of the soft tissue was evident in CFA-induced groups when compared with the paws of unaffected mice. Dexamethasone reduced this soft tissue inflammation. Similarly, the honokiol treated group exhibited marked reduction in inflammation as compared to the negative control group.

### Effect of Honokiol on Histological Analysis

Hematoxylin and eosin staining results showed significant proliferation in the epithelial layer of the hind paw of CFA-induced paw as compared to normal paw (**Figure 13a**). Similarly, abnormal and deeply dense cells were observed in the inflamed area of CFA group as shown in **Figure 13b**. On the other hand honokiol treatment significantly reduced leukocyte infiltration and edematous tissues **Figure 13d**. Dexa (**Figure 13c**) also significantly reduced infiltration and edema.

### Effect of Honokiol on Muscle Coordination Activity

**FIGURE 14:**
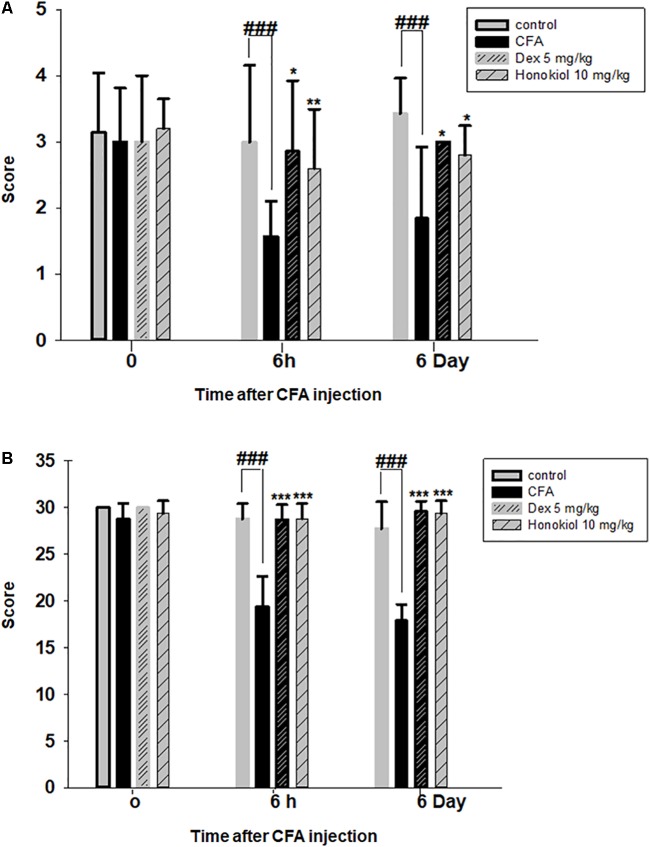
**(A)** Effect of honokiol on CFA induced muscle activity by inverted tray method. The effect on muscle activity was measured 6 h and 6 days after CFA injection, the data are represented as the means SD; (*n* = 5 mice per group) of scoring average. Scoring the inverted screen was done by following method, Scoring the inverted screen was done by the following method, falling between 1 and 10 s = 1, falling between 11 and 25 s = 2, falling between 26 and 60 s = 3, falling after 60 s = 4. **(B)** Effect of honokiol on CFA induced muscle activity by chain weight method. The effect on muscle activity was measured 6 h and 6 days after CFA injection the data is represented as the means SD; (*n* = 5 mice per group) of scoring average. Scoring the weight chain was done by the following method. The number of links in the heaviest chain held for the 3 s, multiplied by the time (s) it is held. If the heaviest weight is fell before 3 s a suitable intermediate value is selected and added to it. ^###^*P* < 0.001 indicates a significant difference from the control group.

To study the potential side effect of honokiol, muscle activity was assessed by using chain method and inverted tray method. It was observed that both at 6 h and 6 days, no significant deleterious effect was observed on muscle activity. Results are shown in **Figures 14A,B**.

### Effect of Honokiol on Gastric Mucosa

**FIGURE 15:**
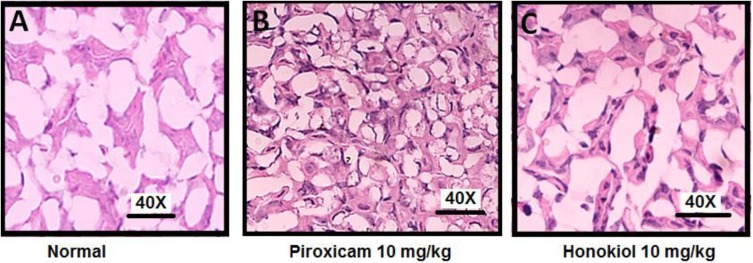
Effect of 3 days, oral honokiol treatment on GIT mucosa by H&E stained tissue sections **(A)** Normal GIT mucosa, **(B)** Piroxicam (10 mg/kg oral) GIT mucosa, **(C)** Honokiol (10 mg/kg oral) GIT mucosa. GIT mucosa were analyzed on day 3 post-treatment. Each section was presented at ×4, ×10, and ×20 original magnifications. In piroxicam multiple arrows indicate the location of distortion of superficial layer of gastric mucosa. Stomach showing desquamation of superficial layer of gastric mucosa. However, no such side effect is being observed in honokiol treated group.

The H&E staining was performed to investigate the effect of oral administration of honokiol on the GIT mucosa. H&E staining when compared with normal GIT mucosa (**Figure 15A**), indicates that honokiol oral treatment is not associated with any toxic effect on GIT mucosa **Figure 15C**. However, the piroxicam treatment exhibited significant toxic effect on GIT mucosa as evident from **Figure 15B**.

## Discussion

Pain is one of the most incapacitating and debilitating condition which is the consequence of a number of diseases or may itself be the cause of discomfort individually. Present treatments available for pain management have numerous side effects causing various problems and there is a need to develop new safe and effective treatments (Kidd and Urban, 2001). In the present study, the anti-nociceptive effect of honokiol on various events and mediators of inflammatory pain were explored in order to gain insight into the mechanism behind the anti-nociceptive effect of honokiol. Then mediators of inflammatory pain were explored in order to gain insight into the mechanism behind the anti-nociceptive effect of honokiol. Honokiol was found effective from 2 h till 6 h, in acute studies. As well as the effect was persistent in chronic model of pain.

Honokiol is a polyphenol, isolated from genus Magnolia. According to the previous finding (Kim et al., 2008), honokiol demonstrated potent anti-inflammatory properties. On the basis of previous findings, it is hypothesized that honokiol reduces pain sensation and may involve various inflammatory signaling pathways. It is a well understood fact that both carrageenan and CFA produce chronic inflammatory pain due to the release of numerous inflammatory mediators, initiating long-term variations in nervous system and immune system (Khan et al., 2016). Therefore, the local injection of CFA results in the augmented release of mediators, such as NO, TNF-α, IL-1β, and IL-6, modulated by NF-κB (Hayden and Ghosh, 2012). Honokiol attenuating CFA-induce pain might be acting on these signaling pathways and exhibit their anti-nociceptive effects (dos Santos et al., 2010). Cytokines are the diverse class of regulatory proteins produced by the white blood and some other cells. Among cytokines, interleukin (IL)-6 is markedly upregulated during pain and hyperalgesia (Sommer and Kress, 2004). Similarly, IL-1β is a vital mediator of inflammatory responses, and plays an important role in many cellular processes like apoptosis and proliferation. IL-1β is the inducer of cyclooxygenase-2 (COX2) and contributes to inflammatory pain hypersensitivity (Herder et al., 2015). The present study significantly revealed that honokiol decreased the production of various cytokines in paw, which is a common effect of polyphenol compounds (Menghini et al., 2016).

Nitric oxide plays an important role in many physiological processes including blood pressure regulation, immune system response, and communication between nervous system components (Khan et al., 2013a). Moreover, it is well recognized as a central mediator and inflammatory regulator (Luiking et al., 2010). The role of nitric oxide in pain and inflammation is very complex because it is considered to have both anti- and pro-nociceptive effects. Nitric oxide contributes to the development of central sensitization. On the contrary, previous studies have also established that NO exhibited anti-nociception in the peripheral and as well as in the central nervous system (Cury et al., 2011). In present study there is decrease in NO levels which may indicates that honokiol act mainly by decreasing inflammation.

Previous studies suggested that the oxidative stress might play a significant role in inflammatory and chronic pain (Vaculin et al., 2010). Oxidative stress is caused by the dysregulations between the production as a side product of normal metabolism and elimination by the cellular defense of the body (Choi et al., 2014). In present study it is demonstrated that honokiol remarkably potentiated the expression levels of antioxidant enzymes, which is supported by previous study (Ohtsuji et al., 2008). In oxidative stress, Nrf2, a basic zipper like leucine (bZIP), is released and binds to the region of phase II detoxifying enzyme HO-1 (Khan et al., 2016). HO-1 is an enzyme that catalyzes the degradation of heme. The degradation products of these enzymes are under special consideration in novel research the diverse pharmacological application including antioxidant (Ogata et al., 1997; Amorati et al., 2015), anti-inflammatory and cell protective effects (Deshane et al., 2005). Moreover, induction of HO-1 occurs as an adaptive and beneficial response to several injuries. The beneficial role of HO-1 has been associated with numerous clinically related diseases involving multiple body systems as well as important biological progressions such as ischemia-reperfusion injury, inflammatory diseases and transplantation. HO-1 may therefore be a crucial target molecule for some ailments like pain (Deshane et al., 2005).

Vascular endothelial growth factor is widely known to have a regulatory effect on blood vessel function and integrity (Shibuya, 2014). However, any change in the VEGF signaling and expression which is strongly related to various angiogenic related diseases associated with the development of chronic pain (Hulse, 2017). VEGF acts on sensory neurons by inducing their outgrowth and survival (Nesic et al., 2010). Furthermore, VEGF is also found to be essential for the modulation of chronic pain (Hulse, 2017). Honokiol was shown previously to block autophosphorylation of the VEGF receptor (Wen et al., 2009). In the present study, the expression of VEGF decreases significantly by honokiol which further supports the anti-nociceptive potential of honokiol. Similarly, the most commonly used analgesics and conventional NSAIDS have deleterious effects on gastro intestinal track (GIT) due to their topical irritating effect, activation of neutrophil and reduced production of PGEs (Wallace, 2000). The gastro-protective effect of honokiol was already established in previous reports (Chan et al., 2008). In the current study, the histological analysis of stomach mucosa demonstrated that the honokiol didn’t produce macroscopic mucosal damage similar to that observed with piroxicam.

To explore the possible mechanism of action of honokiol, various types of agonist and antagonists were selected for further study. The anti-nociceptive role of NSAID is well established, suggesting the possible involvement of inflammation in the process of nociception (Long et al., 2016). Similarly, the opioid receptor agonist also showed pain reducing potential (Moulin et al., 2002). Anticonvulsants and antidepressants drugs are also well known targets of pain control regimens (Jackson, 1998). On the basis of this principle, the involvement of these pathways in the anti-nociceptive potential of honokiol was further assessed. This study demonstrated that the co-administration of honokiol with piroxicam showed remarkable synergism suggesting that honokiol acts on inflammatory cytokines and cyclooxygenase. Similarly, the augmented effect of tramadol on honokiol anti-nociceptive potential suggests that honokiol may interfere with opioid and monoamine reuptake system. The activation of opioid receptor in Aσ and C fibers inhibits the calcium channel with resultant decreases in cAMP levels (Wang et al., 2015). Decreased cAMP levels then prevents the release of substance P, cGRP, and glutamate (Leppert, 2009), which is consistent with a previous study which inferred that honokiol inhibits the release of these mediators (Liou et al., 2003). Partial blocking of honokiol anti-nociceptive effect through naloxone further strengthen this hypothesis. The co-administration of GABA agonist and antagonists with honokiol interfere with pain significantly, thus suggesting that honokiol may interact with voltage gated Ca^+^ system for its anti-nociceptive effects (Kukkar et al., 2013). Based on these results, the present study demonstrated that the GABA antagonist (flumazenil) was capable of completely interfering while opioid antagonist (naloxone) was observed that it might be capable of partially interfering with the anti-nociceptive effect of honokiol, suggesting that honokiol might interact with these systems (upstream or downstream) to control pain. By and large, honokiol observed to increase the pain threshold, and also showed comparable improvement in biochemical parameters indicating their possible role in the management of pain.

## Conclusion

The present work provides substantial evidence that honokiol produces significant anti-hyperalgesic and anti-allodynic effects in both acute and chronic pain models induced by CFA and carrageenan in various animal models. It also attenuates the pain induced by thermal hyperalgesia. Furthermore, honokiol also significantly reduced CFA and carrageenan induced pro-inflammatory cytokines mRNA expressions. Genes linked to antioxidants were observed to upregulated by the treatment of honokiol. Honokiol seems to be reasonably safe as results of liver function test and muscle activity were in line with positive control dexamethasone. Therefore, the present study shows that honokiol might be a potential candidate for further development as a treatment option for acute and chronic pain.

## Author Contributions

All authors listed have made a substantial, direct and intellectual contribution to the work, and approved it for publication.

## Conflict of Interest Statement

The authors declare that the research was conducted in the absence of any commercial or financial relationships that could be construed as a potential conflict of interest.

## Abbreviations

CFA: complete Freund’s adjuvant
CREB: cAMP responsive element binding protein
Dexa: dexamethasone
GAPDH: glyceraldehyde 3-phosphate dehydrogenase
GIT: gastrointestinal tract
H&E staining: hematoxylin and eosin staining
HO-1: heme oxygenase
i.p.: intraperitoneal
i.pl.: intraplantar
MAPKs: mitogen-activated protein kinases
NF-κκB: nuclear factor-κκB
NO: nitric oxide
Nrf2: nuclear factor erythroid 2–related factor 2
qRTPCR: quantitative Real Time PCR
SOD2: superoxide dismutase
VEGF: vascular endothelial growth factor
